# Elementary Chemistry, Theoretical and Practical

**Published:** 1850-12

**Authors:** 


Art. VIII.—*Elementary Chemistry, theoretical and practical.* By George Fownes, F. R. S., Professor of Practical Chemistry in University College, London. Edited, with additions, by Robert Bridges, M.D., Professor of Chemistry in the Philadelphia College of Pharmacy, etc., etc. Third American, from a late London edition. With numerous wood engravings. Philadelphia: Lea & Blanchard. 1850.

The high scientific character of the work of the late Mr. Fownes has made it one of the established favorites among those who study chemistry. The author was one of the most laborious, earnest, and energetic investigators in those fields, wherein Liebig has won renown, and even in the blaze of light that surrounds that great name, the reputation of Fownes shines with no inferior luster. He devoted his life to the science which he loved and adorned, and up to within a few hours of his death, he was engaged in labor upon the work before us. His mind was remarkably well adapted for chemical science, and no one ever devoted himself to it with more ardor and fervency than Mr. Fownes. His "Chemistry for Students" is one of the most concise, clear, and valuable works of the kind we have ever read. A

student who cannot understand the work of Fownes, may as well surrender all hopes of chemistry at once—he may consider it a sealed volume to him.

The work of Mr. Fownes was not designed for a manual of chemistry, it is intended for beginners in the science, and he leads those who follow him properly through paths of light. One of the most eloquent of the eulogists of Robert Burns, says, "the poor man climbed with difficulty the hill, on which the Temple of Knowledge stands, but the son of genius, had not a key that would open any one lock, in the spacious building." No one, who may desire a knowledge of chemistry need sink under that want, while he can command such a key as is furnished in this book. There is no elementary work on chemistry that contains a finer body of information on "Heat," "Light," "Magnetism," and "Electricity." When we look back upon what was taught on these subjects twenty years ago, and compare it with the teaching of the present time, it is difficult to recognise the subjects, except in their names.

A great variety of matter has been introduced into this edition, which, on account of the progressive nature of the science was not in any of the former editions. Organic Chemistry, under the investigating labors of its devotees, is constantly augmenting its volume of facts, and shedding new light upon various subjects. This is the department of chemistry that is of special interest to those who cultivate practical medicine, for the purpose of advancing it, and we commend such persons to that department of Mr. Fownes's work.

The American edition is a beautiful volume, it is well printed and illustrated, and deserves the confidence and patronage of the profession.

